# Reduction in microbial survival on food contact surfaces by a spray coated polymerized quaternary ammonium compound

**DOI:** 10.1002/fsn3.1537

**Published:** 2020-03-24

**Authors:** Jaesung Lee, Melvin A. Pascall

**Affiliations:** ^1^ Department of Food Science and Technology Ohio State University Columbus OH USA

**Keywords:** antimicrobial activity, *Escherichia coli*, food contact surface, *Listeria*, surface sanitization

## Abstract

Using polymerization and immobilization techniques, the loss of antimicrobial efficacy of a quaternary ammonium compound (QAC) was minimized by decreasing its solubility and crosslinking it to metal substrates. The survivability of *Listeria innocua* and *Escherichia coli* K12 inoculated to silane QAC coated metal surfaces was compared with uncoated metal surfaces at different treatment conditions for up to 6 months storage. Resilience of the coating material to repeated cleaning, up to 20 washing and rinsing cycles, was also investigated. No evidence of bacteria viability (>5 log reduction of colony‐forming unit) was observed for *L. innocua* when they were inoculated onto coated surfaces stored for 3 months, whereas *E. coli* was reduced by 3 to 4‐logs. For the viable *L. innocua* cells on the coated surfaces, >5 log reductions were achieved even after the coated surfaces were cleaned by 20 washing and rinsing cycles prior to the cells’ inoculation. For the *E. coli* cells, ~ 2 log reductions were achieved after 5 cleaning cycles and <1 log reduction after 10 or more cleaning procedures. Overall, the results showed that the coating had antimicrobial activity against Gram‐positive bacteria while it showed moderate activity to Gram‐negative bacteria.

## Graphical abstract

This study investigated the ability of a quaternary ammonium compound to adhere to a metal surface and resist several attempts to diminish its antimicrobial efficacy after several washing and rinsing cycles during a six‐month period. The antimicrobial compound was more effective against Gram‐negative bacteria, when compared with Gram‐positive bacteria

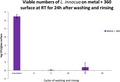

## 1 INTRODUCTION

Cross‐contamination with undesirable microorganisms during food preparation was identified in many reports as a major factor associated with foodborne illnesses and spoilage. In food processing facilities, reduced survivability of these organisms on food contact surfaces would help to mitigate the risk of subsequent microbial transfer (Kusumaningrum, Riboldi, Hazeleger, & Beumer, 2003; Podolak, Enache, Stone, Black, & Elliott, 2010). Therefore, various chemical sanitizing agents have been used to prevent the spread of microbial contaminants on various food contact surfaces. Among those sanitizing agents, oxidizing chemicals such as hypochlorites, chlorine dioxide, peracetic acid, hydrogen peroxide, and glutamine‐derived aldehydes are widely used on food preparation surfaces since they strongly inactivate microorganisms by inducing chemical oxidation of the cellular components in the organisms (Cortezzo, Koziol‐Dube, Setlow, & Setlow, 2004). Used at the correct concentration/time/temperature combination on food contact surfaces with moderate organic matter and bacterial loads, these oxidizing agents have shown the ability to reduce bacterial numbers to levels acceptable to the food processing industry. However, some of these agents are corrosive to equipment at the concentrations required for microbial inactivation and they produce harmful and/or unknown byproducts when they cause the oxidation of organic matter. Additionally, studies have showed that these chemicals have the potential to produce carcinogenic free radicals (Bull & Cotruvo, 2014; Langlais, Reckhow, & Brink, 1991). Finally, these modes of inactivation have limited residual efficacy and therefore must be routinely applied to surfaces to obtain desired effects and reduce overall contamination. On the other hand, sanitizing agents containing quaternary ammonium compounds (QAC) are relatively safe within prescribed limits and cause no problems to humans if used as per the Environmental Protection Agency (EPA) recommendations. Unlike oxidizing chemicals, QAC are solid compounds that can be easily dissolved, and they do not readily evaporate. Therefore, QAC are considered to be an excellent candidate for the polymerization of antimicrobials which have prolonged activity. Additionally, since the cellular membranes of most bacteria are negatively charged, they are very sensitive to cationic sanitizers such as QAC (Chen & Cooper, 2002; Blustein, Hinkle, & Smith, 2005).

On food contact surfaces, the efficacy of an antimicrobial agent incorporated into a biopolymer is mainly related to the good film‐forming property of the polymer and its ability to sequester and subsequently release the antimicrobial agent. Many techniques can be used to attach antimicrobial polymers to food contact surfaces using electrostatic or exclusion steric repulsion mechanism, and thus prevent microbial cells from attaching to these surfaces (Kougia et al., 2015; Tiller, 2010). However, since major microbial contamination in food processing facilities occurs from food sources, organic matter such as food particles has helped to shield contaminating bacteria from the direct repelling activity of antimicrobial biopolymers. Therefore, using innovative polymerization and immobilization techniques, our study was conducted to preserve the antimicrobial efficacy of a sanitizing agent by decreasing its solubility by crosslinking and binding it to food contact surfaces. The active ingredient was 3‐trihydroxysilyl‐propyldimethyloctadecyl ammonium chloride, and the active antimicrobial agent was a silane quaternary ammonium (silane QAC) salt. Therefore, the actual structure was comprised of silane, nitrogen, and carbon atoms. The ingredients in our tested silane QAC were certified by the EPA (2018) and approved by the FDA to be used on direct food contact surfaces. The surface coating was developed and applied as a silane QAC salt in a spray format. It was an electrostatic spray application which atomized and applied a positive charge to the silane QAC droplets as they came out of the nozzle of the sprayer. Measuring 30–60 microns in diameter, these droplets were roughly 900 times smaller than those produced by conventional sprayers. This positive charge allowed the solution to create a cohesive 360‐degree coverage on the targeted substrates, which upon drying, created a long‐term resilient coating on the surface. This unique bonding characteristic allowed the silane/QAC complex to remain on the surface even under harsh conditions.

The main object of this study was to investigate the growth and/or survival of foodborne microorganisms transferred to a coated, when compared with an uncoated metal surface at different environmental conditions. The antimicrobial resilience of the coating material to repeated cleaning procedures, up to 20 washing and rinsing cycles, was also studied. *Listeria innocua* (Gram‐positive bacteria) and *Escherichia coli* K12 (Gram‐negative bacteria) were tested as surrogates for *Listeria monocytogenes* and *Escherichia coli* O157:H7, respectively. The use of surrogates has been proven to be a practical alternative for foodborne pathogen testing (Moce‐Livina, Muniesa, Pimenta‐Vale, Lucena, & Jofre, 2003).

## 2 MATERIALS AND METHODS

### 2.1 Metal surface coating application

The test metal surfaces were 5.0 × 2.5 cm pieces of Type 302 stainless‐steel sheets for testing and storage at room temperature (23°C). One set of the metal sheets received one coat of the silane QAC and the other set was uncoated. For the coated samples, once the metal surfaces were thermally sterilized and cooled, the QAC solution was applied at the MicroShield 360 facilities and allowed to dry for thirty minutes prior to storage. The average thickness of the coated layer on each metal sheet was <5 nm. The samples were then stored at 23°C for up to 6 months.

### 2.2 Bacteria preparation

*Listeria innocua* Seeliger (ATCC 33090) and *Escherichia coli* K12 (ATCC 29181) cells were separately grown on Tryptic soy agar (TSA) slants and then transferred to Tryptic soy broth (TSB), followed by incubation at 37°C for 21 hr. The final cell concentrations in the broth were 9–10 log colony‐forming units (CFU)/ml. The cells were then harvested by centrifugation (Sovall^®^ RC5C Plus, Newtown, CT) at 10,000 g for 10 min at 4°C. The supernatant was decanted, and the pelleted cells were re‐suspended in 20 ml of sterile phosphate buffer solution (PBS, pH 7.2) to obtain viable cell populations of approximately 9 log CFU/ml. The cell suspensions were serially inoculated into sterile PBS and mixed to give the desired initial numbers.

### 2.3 Antimicrobial activity testing

An industrial antimicrobial method (ISO 22196) was used, with modifications, to evaluate the efficacy of the test metal sheets. At each testing period (after 0, 3, and 6 months of storage), each metal sheet was placed in separate sterile petri dishes with the test surface facing upwards. A 0.1 ml aliquot of bacterial cell suspension (~10^6^ CFU/ml) in a 1/500 dilution of nutrient broth was inoculated onto both metal sheet surfaces (coated and noncoated). Each sample was covered with a piece (20 mm × 20 mm) of sterilized low‐density polyethylene (LDPE) film (10‐µm thick) obtained from Central Ohio Bag & Burlap Inc. and gently press down so that the bacterial inoculum was made to spread to the edges of the sample. All samples were stored in sealed jars at 23 ± 1°C (RT) and cold temperature (10 ± 1°C, CT) at ~95 **±** 2% relative humidity (RH) for 24h. Hygiene cotton swabs obtained from Puritan Medical Products (Guilford, Me) were used to collect the organisms on the surfaces. These swabs were used to transfer microorganisms to test tubes containing 2 ml of peptone water (0.1%) and universal neutralizing chemicals (0.07% lecithin, 0.5% Tween 80, 0.5% NaCl, and 0.1% sodium thiosulfate) (Sigma‐Aldrich). After suspending in the tubes, the cells were serially diluted and plated onto tryptic soy agar to determine bacterial cell viable counts after 24 to 36 hr incubation at 37°C.

### 2.4 The resilience of the coating to repeated cleaning

After two months of storage, one set of coated metal sheets was cleaned by washing [horizontally for three times + vertically for three times by scrubbing with a heavy duty sponge (Scotch‐Brite^®^)] in a washing sink containing soft tap water (43°C, pH ~ 7.9) containing 100 ppm Monsoon detergent (Ecolab Inc.), rinsed for 10 s by dipping into fresh soft tap water, then air‐dried for 1 hr. The process was repeated up to 5, 10, 15, and 20 times prior to the bacterial inoculation. Following the bacterial enumeration procedures described in the antimicrobial activity testing section, the efficacy of the coated sheets was compared with the result obtained for the unwashed (0 cycle) coated sheet.

### 2.5 Microbiological and statistical analysis

The detection limit for the test organisms was 2 CFU/surface for the hygiene swab method. No less than three replicates were tested in each experiment. Variances of the microbial viability were analyzed by the general linear model function and Tukey's comparison testing with Minitab 16 (State College, PA) to determine the level of significance (*p* < .05).

## 3 RESULTS AND DISCUSSION

### 3.1 Survival of the microorganisms on the coated metal surfaces

**Figure 1:**
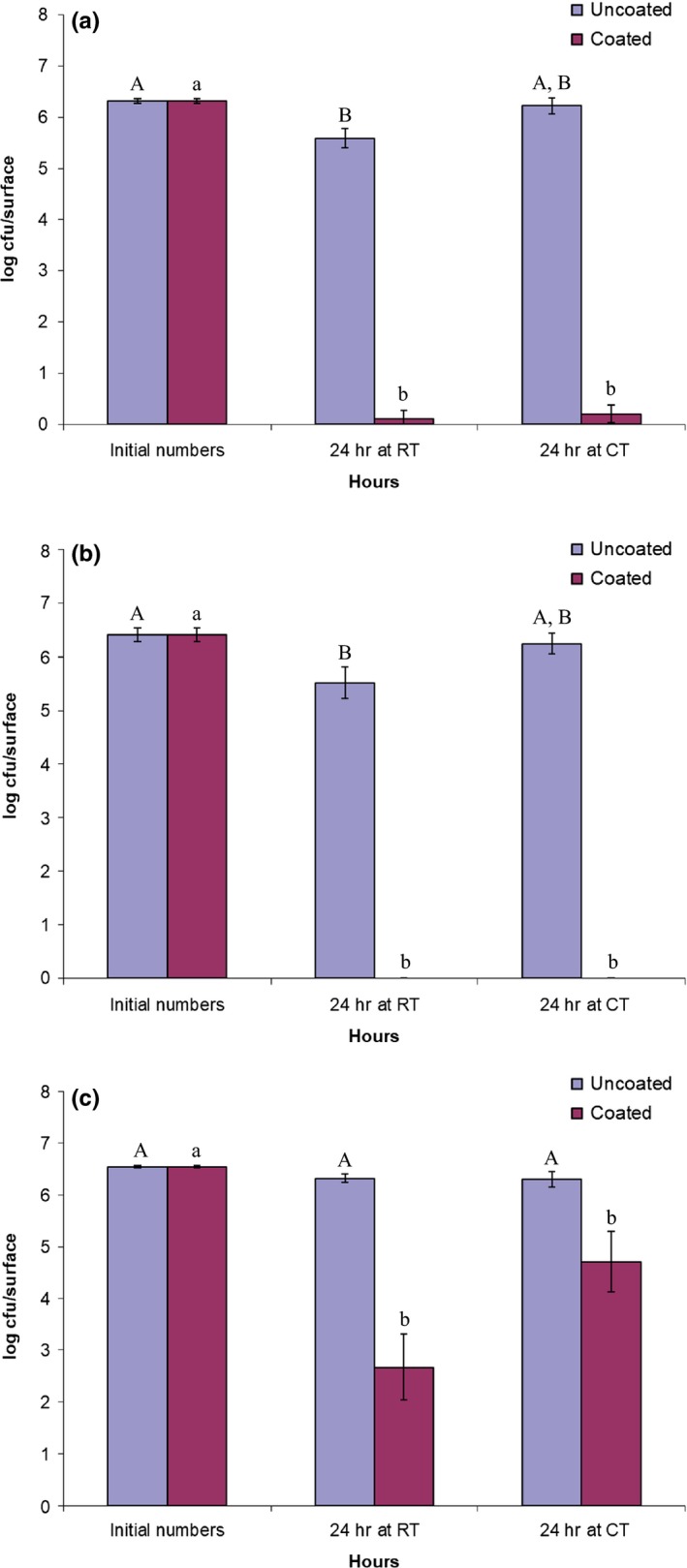
The numbers of viable cells of *L. innocua* on the test surfaces during 24 hr treatment at room temperature (23°C, RT) and cold temperature (11°C, CT) after storage for 0 month (a), 3 months (b), and 6 months (c). Means for the reduction of *L. innocua* on uncoated surface (A and B). Means for the reduction of *L. innocua* on coated surface (a and b). Values with the same letter are not significantly different (*p* > .05)

**Figure 2:**
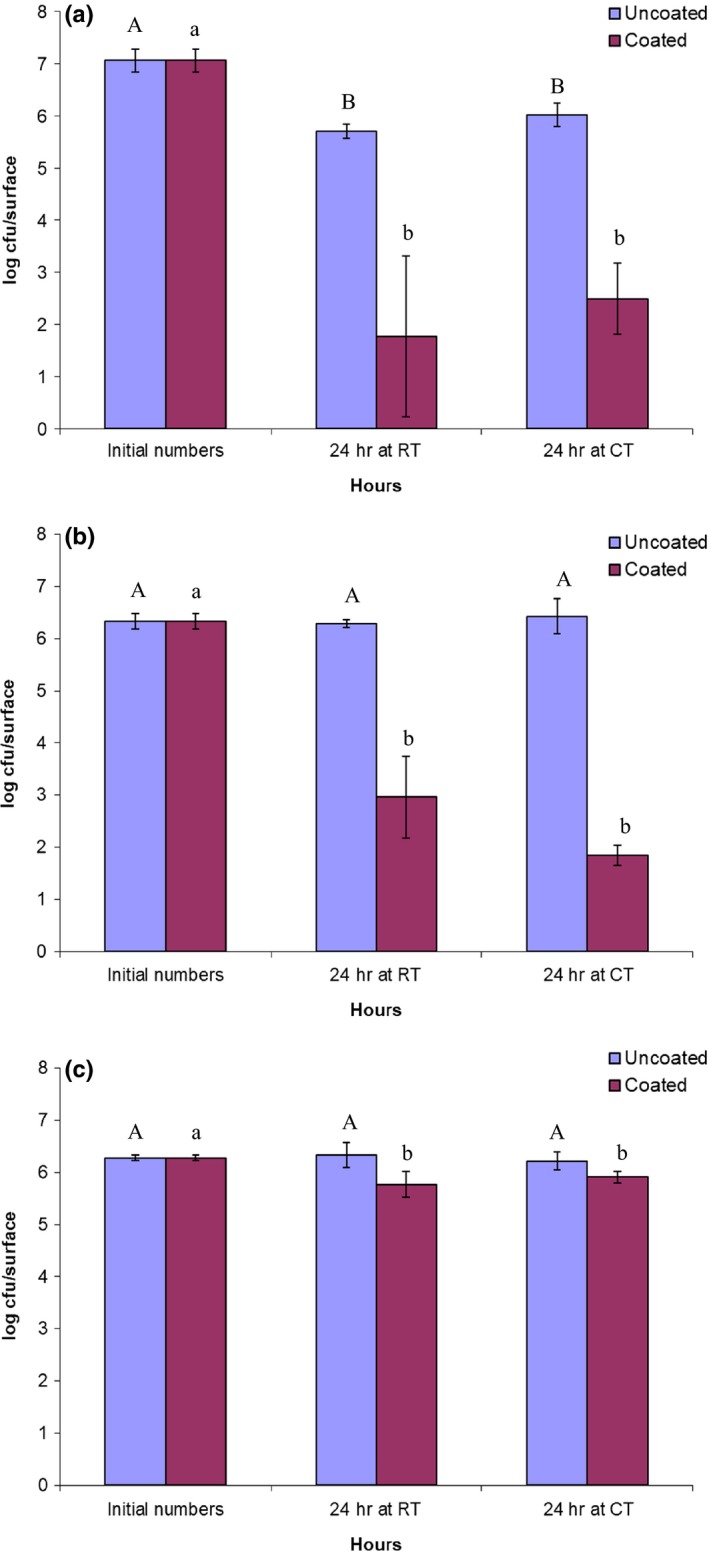
The numbers of viable cells of *E. coli* K12 on the test surfaces during 24 hr treatment at room temperature (23°C, RT) and cold temperature (11°C, CT) after storage for 0 month (a), 3 months (b), and 6 months (c). Means for the reduction of *E. coli* K12 on uncoated surface (A and B). Means for the reduction of *E. coli* K12 on coated surface (a and b). Values with the same letter are not significantly different (*p* > .05)

The effectiveness of the sprayed QAC coating material for the reduction of viable bacterial cells attached to the metal sheets is shown in Figure 1 for *L. innocua* and in Figure 2 for *E. coli*. After less than a week (0 month) of storage at room temperature (23°C) for the sprayed metal sheets, almost no evidence of the bacterial viability (>5 log reduction of CFU) was observed for *L. innocua* when the cell suspension was inoculated onto the coated surface for 24 hr. For *E. coli,* it was reduced by 3 to 4‐logs. It is generally believed that impermeability of the outer membrane in the cell wall of Gram‐negative bacteria, such as *E. coli*, does not allow many types of chemical agents to reach the inner cytoplasmic membrane (Sundheim, Langsrud, Heir, & Holck, 1998). However, similar studies by Li et al. (2015) and Kougia et al., (2015) showed significant reductions in Gram‐negative and Gram‐positive bacteria. This shows that there is potential for this polymerization and immobilization technique of QAC compounds for antimicrobial applications in environments where there are concerns about microbial cross‐contamination. Previous studies by other researchers also observed that Gram‐negative bacteria were less sensitive than Gram‐positive bacteria when QAC based sanitizers were applied to inactivate microbial contaminants on the surfaces of various table‐ware items (Handojo, Lee, Hipp, & Pascall, 2009; Lee, Cartwright, Grueser, & Pascall, 2007). The viable cell numbers of the inoculum on the uncoated (control) surfaces remained the same after each test trial for both organisms. Also, there was no significant difference (*p > *.05) in the viability of the microbial numbers found on the coated surfaces after been treated for 24 hr at cold (10°C, CT) and room temperature (23°C, RT) conditions for each bacterial strain. This indicated that the antimicrobial compounds in the coating were not significantly weakened at the cold testing temperature. Previous food preservation studies have also shown that the antimicrobial activities of coating materials based on organic chemical compounds were not affected by relatively low ambient temperatures (<10°C) that are maintained at some food processing and preservation facilities (Campaniello, Bevilacqua, Sinigaglia, & Corbo, 2008; Sogvar, Saba, & Emamifar, 2016).

When the coated metal sheets were tested after three months storage, their efficacies for cell reductions of both bacterial stains remained the same as the results obtained from the metal sheets at 0‐month storage (Figure 1a,b; Figure 2a,b). Although the mean reductions of the *E. coli* cell viability were slightly greater than the reductions obtained at 0‐month storage, the disparities were not significant (*p* > .05). The results suggested that although the thickness of the coated layer was thinner than that of conventionally sprayed coatings, the active compounds remained on the surface without loss of their antimicrobial properties during the three‐month storage period.

Six months of storage for the coated samples significantly lowered the antimicrobial properties of the compounds against both bacterial strains. It was shown that after treatment for 24h, less than a two log reduction of the *L. innocua* viability was achieved for the samples tested at CT condition, while ~4 log reductions were observed at RT condition (Figure 1c). The results implied that temperature conditions at testing can be a significant factor when longer term storage of coated samples are to be used for psychrotrophs or cold‐tolerant microorganisms like *L. innocua*. For the *E. coli, <*1 log reduction (<90% reduction) was observed on the six‐month‐old coated surfaces at both testing temperatures.

### 3.2 Determination of the resilience of the coating material to repeated cleaning procedures

**Figure 3:**
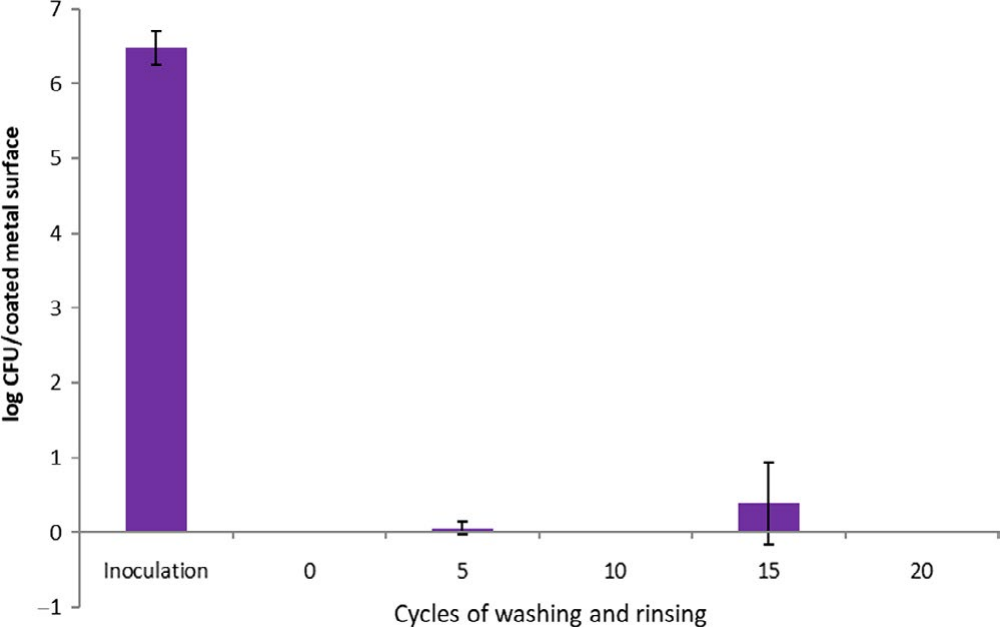
The numbers of viable cells of *L. innocua* on the treated test surfaces exposed to a total of 20 wash and rinse cycles

**Figure 4:**
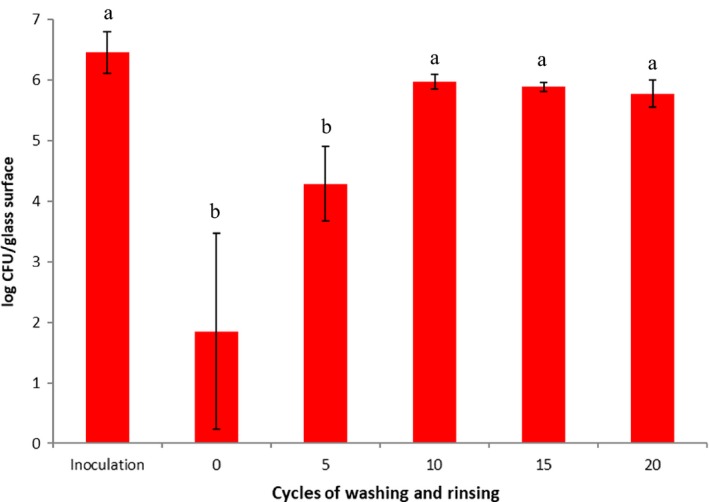
The numbers of viable cells of *E. coli* K12 on the treated test surfaces exposed to a total of 20 wash and rinse cycles. Means for the reduction of *E. coli* K12 on coated surface (a and b). Values with the same letter are not significantly different (*p* > .05)

The typical manual‐cleaning procedure (washing, rinsing) was used to clean the coated metal sheets at each cleaning cycle. This procedure has been generally applied in food preparation facilities to remove undesirable organic matters on food contact surfaces prior to sanitation (Handojo et al., 2009; Lee et al., 2007). The bacterial survivabilities after repeated cleaning procedures on the coated metal surfaces (without additional spray) are shown in Figure 3 (for *L. innocua*) and Figure 4 (for *E. coli*), respectively. For the viable *L. innocua* cells on the coated surfaces, very small numbers were detected even after the coated metal surfaces were cleaned by 20 washing and rinsing cycles. On the other hand, the *E. coli* cells were not affected much (<1 log reduction) by the coated surfaces after 10 or more cleaning procedures were applied.

As can be seen in the first part of this study, *E. coli* showed much stronger resistance to the coating compounds during each cleaning cycle when compared with *L. innocua*. The results are not surprising since Gram‐negative bacteria (such as *E. coli* strains) are protected by an additional cell membrane called “The outer membrane (OM)” which acts as an intrinsic barrier to hydrophobic antimicrobial agents such as QAC (Bore et al., 2007; Helander et al., 1998). Since many spoilage and potential pathogens in food processing facilities belong to the Gram‐negative bacteria group, many studies have been conducted on how to best destroy or disintegrate their OM structure. In the OM structures, lipopolysaccharide (LPS) molecules in the outer leaflet of the membrane are considered to create resistance to hydrophobic antimicrobial agents (Nikaido, 2003). The LPS is composed of three parts: lipid A, O‐antigenic polysaccharides, and a core oligosaccharide. Previously, studies were conducted to weaken the OM using permeabilizers, which induced damage to the LPS layer. This then allowed an increase in the permeability of the OM to hydrophobic agents. In the use of mechanical methods, Lee and Kaletunç (2010) used high hydrostatic pressure for mechanical disintegration of the OM of Gram‐negative *Salmonella enteritidis,* then observed increasing sensitivity to nisin. They also examined the disintegration of the LPS component of the bacteria using differential scanning calorimetric (DSC) analysis. In the case of chemical permeabilizers, compounds such as EDTA have shown promising results. Also, undissociated forms of weak acids such as benzoic, citric, lactic, sorbic, and acetic acids have been considered as good permeabilizers against Gram‐negative bacteria since the undissociated forms of the acids can pass through both outer and inner cell membranes and consequently lead to cell death (Hirshfield, Terzulli, & O’Byrne, 2003).

The next phase of our study will be to reformulate the coating material to incorporate either chelating agents such as EDTA or weak acids, so that it could be more effective in inhibiting the growth of Gram‐negative bacteria such as *E. coli*.

## 4 CONCLUSION

Overall, the results of our study showed that the silane QAC coating technology has antimicrobial activity against Gram‐positive bacteria even in the presence of repeating washing while it showed moderate activity to Gram‐negative bacteria. Since Gram‐negative bacteria are protected by an additional cell membrane (called the outer membrane) against chemical antimicrobial agents, it could be possible to modify the technology by using an alternative chemical approach. This approach is currently being developed. Importantly, the proven residual protection provided by the tested silane QAC coating technology adds protection between commonly used cleaning and sanitizing methods discussed above in the introduction. As such, the range of results between both Gram‐positive and Gram‐negative are still better than having zero protection between cleanings.

## CONFLICT OF INTEREST

The authors declare that they do not have any conflict of interest.

## ETHICAL STATEMENT

Ethical Review: This study did not involve any human or animal testing. Informed Consent: Written informed consent was obtained from all study participants prior to submitting the manuscript for publication.
